# PMDedu: Assessing the educational needs of startups and academic investigators focused on pediatric medical device development

**DOI:** 10.1017/cts.2023.633

**Published:** 2023-09-25

**Authors:** Payal Shah, Alexis Snitman, Jennifer McCaney, Lynn M. Rose, David Sheridan, Juan Espinoza Salomon

**Affiliations:** 1 Department of Pediatrics, Children’s Hospital Los Angeles, Los Angeles, CA, USA; 2 University of Southern California, Los Angeles, CA, USA; 3 Department of Decisions, Operations and Technology Management, University of California Los Angeles, Los Angeles, CA, USA; 4 Department of Pharmacy, University of Washington, Seattle, WA, USA; 5 Department of Emergency medicine, Oregon Health & Science University, Portland, OR, USA; 6 Stanley Manne Children’s Research Institute, Ann & Robert H. Lurie Children’s Hospital of Chicago. Chicago, IL, USA; 7 Northwestern University Feinberg School of Medicine, Chicago, IL, USA

**Keywords:** Pediatric medical device, Innovators, startups, investigators, FDA regulation, education assessment

## Abstract

**Background:**

The pediatric medical device development (PMDD) process is highly complex, beset by a variety of financial, technical, medical, and regulatory barriers. Startup company innovators and academic investigators often struggle with accessing specialized knowledge relating to regulatory requirements, product development, research, and marketing strategies.

**Objectives:**

The West Coast Consortium for Technology & Innovation in Pediatrics (CTIP) conducted an educational needs assessment to understand knowledge gaps and inform our educational strategy.

**Methods:**

We surveyed a total of 49 medical device startups and 52 academic investigators. Electronic surveys were developed for each group on Qualtrics and focused on manufacturing, regulatory, research, commercialization, and funding. Descriptive statistics were used.

**Results:**

A larger proportion of academic investigator respondents had a clinical background compared to the startup respondents (45% vs. 22%). The biggest barriers for academic investigators were understanding regulatory and safety requirements testing (52%) and finding and obtaining non-dilutive funding was the most difficult (54%). Among startups, understanding clinical research methods and requirements was the biggest barrier (79%).

**Conclusion:**

Startup companies and academic investigators have similar, but not identical, educational needs to better understand the PMD development process. Investigators need more support in identifying funding sources, while startup companies identified an increased need for education on research regulatory topics. These findings can help guide curriculum development as well as opportunities for partnerships between academia and startups.

## Introduction

Pediatric medical devices treat or diagnose diseases and conditions from birth through age 21 [1]. The pediatric medical device market accounted for only $25.9 billion compared to the global medical device market size $432.23 billion in 2020 [2,3]. This difference in market size reflects overall resource allocation as well as a number of pediatric-specific barriers to medical device development. Children have unique medical device needs and differences in size, function, anatomy, and physiology compared to adults [4]. To compensate for this gap, adult devices are often adapted or configured to address unmet needs in children, even when there is a lack of safety data in children. Despite new regulatory and legislative initiatives, the percentage of novel approved devices is still stagnant [5]. The lack of devices designed, evaluated, and approved for pediatrics not only limits access to potentially beneficial novel devices but also leads to off-label use of adult devices, potentially altering the risk-benefit profile [6,7].

The US Food and Drug Administration (FDA) aims to motivate industry to enter, sustain, and innovate in the pediatric medical device [6]. These efforts include initiatives like the Pediatric Device Consortia (PDC) grant [8,9], the Humanitarian Use Device (HUD)/Humanitarian Device Exemption (HDE) pathway, the System of Hospitals for Innovation in Pediatrics – Medical Devices (SHIP-MD) program [10], collaboration on the National Evaluation System for health Technology and incorporating Real-World Evidence generation strategies. Education is a critical component of encouraging pediatric medical device innovation. Clinicians, innovators, and medical device manufacturers need to be aware of the unique requirements and barriers that impact pediatric devices [7,11]. The West Coast Consortium for Technology & Innovation in Pediatrics (CTIP) [12] is one of the FDA-funded PDCs, and advances pediatric medical device development through networking, guidance and advising, education, research, advocacy, and non-dilutive funding. In order to better target our educational activities, CTIP conducted an educational needs assessment among various stakeholders from industry and academia to assess their knowledge about pediatric device development, identify gaps, and describe barriers they encounter along the way.

## Methods

The study was exempted and approved by IRB of Children’s Hospital Los Angeles (CHLA) (CHLA-20-00135).

### Startup survey

CTIP conducted the survey for medical device startup companies between July 2020 and February 2021. The online survey was built in Qualtrics [13] and was promoted to pediatric medical device startups through email, social media, and the CTIP website. The survey link contained a research information sheet where the purpose of the study, population, timeline, and methods were described. The survey itself contained 32 items in four sections: 1) Demographics about their company, role, and professional background; 2) Details about their device, device classification, current stage from Total Product Life cycle (TPLC) [14], regulatory submission, and the classified clinical population and appropriate age category; 3) understanding their approach towards learning about pediatric medical devices, preferred resources, and encountered barriers; and 4) What topics they would like to receive educational resources from CTIP. The device classification and TPLC description are provided in Supplemental Table 1.

### Investigator survey

A second survey focused on the perspective of investigators at academic institutions was also built on Qualtrics and sent out in February 2021 via email to investigators at University of Southern California and Children’s Hospital Los Angeles (CHLA), University of California Los Angeles, Oregon Health & Science University, and University of Washington. The survey included 12 items and was divided into three sections: 1) Investigator demographics including academic position or rank, professional background, if they have been involved in PMDD, and if their research led to an invention disclosure, patent, IP or commercialization opportunity; 2) what resources they use to learn more about the PMDD process, barriers they faced in early stages of PMDD versus late stages of PMDD; and 3) how useful they thought additional resources might be to them and other investigators.

### Analysis

The startups and investigators were administered only one survey based on their expertise. We performed the descriptive statistics directly output from Qualtrics from both surveys. To compare survey outcomes, we developed a conceptual map of the questions in each survey and compared the results within the same concept. The full text of both surveys is included in supplemental material “PMDedu startup and investigator survey.”

## Results

### Startup survey

A total of 49 eligible companies responded to the Startup Survey. Demographics of respondents and device categories are in Table 1. Out of the 49 respondents, 38 (78%) of them were company founders and 26 (53%) had an executive position (e.g., chief executive officer, chief operating officer, etc.). Of the professional backgrounds of the participants, 20 (41%) had research and 17 (35%) had an entrepreneur background. 20 (41%) of respondents were women and 11 (22%) reported to identify as a person of color and/or as an underrepresented minority in STEM. 12(24%) respondents were associated with an academic institution and 24(49%) respondents were familiar with the FDA PDC program. 13(27%) companies were a member of medical device advocacy or industry group. On average, respondents represented companies that had existed for 4.6 ± 0.5 years and had an average of 3 full-time employees and 3 part-time employees. 23 out of 49 respondents said they were slightly or not at all familiar with the Total Product Life Cycle. 53% of companies had medical devices that were either in the prototype or advanced prototype stage of development, while only 20% of devices were in the clinical or preclinical stage and 10% in the commercial use stage. A majority (55%) of devices were anticipated to be classified as Class II medical devices; 8% of companies had not determined an anticipated device class. At the time of completing the survey, 63% of respondents had not yet received an FDA regulatory decision or designation. Only 5(10%) devices were considered Software as a Medical Device (SaMD).

**Table 1.** Description of startup survey participants and their pediatric medical devices products

Category (respondents)		*n*	%		Category (Devices)		*n*	%
*Role in company*					*Device Stage*			
	Founder	38	78%			Concept	2	4%
	Inventor	23	47%			Prototype	10	20%
	Executive position	26	53%			Advanced prototype	13	27%
	Researcher	20	41%			Manufacturing	3	6%
	Engineer	11	22%			Preclinical	8	16%
	Other	4	8%			Clinical	2	4%
*Background*						Commercial use	5	10%
	Engineer	16	33%		*Device class*			
	Nurse	2	4%			Class I	9	18%
	Physician	6	12%			Class II	27	55%
	Entrepreneur	17	35%			Class III	2	4%
	Researcher	20	41%			Class II exempt	5	10%
	Allied Health Professional	3	6%			CLIA-regulated product	1	2%
	Other	11	22%			Combination product	1	2%
						To be determined	4	8%
					*Pediatric sub-population*			
						Newborn/Neonate (birth to 1 month)	11	22%
						Infant (>1 month to 2 years)	18	37%
						Child (>2 to 12 years)	29	59%
						Adolescent (>12 through 21 years)	33	67%
					*Regulatory decision from FDA*			
						Exempt device	7	14%
						510(k) clearance	4	8%
						De Novo device classification	3	6%
						Breakthrough device designation	1	2%
						none of the above	31	63%

**Figure 1.**
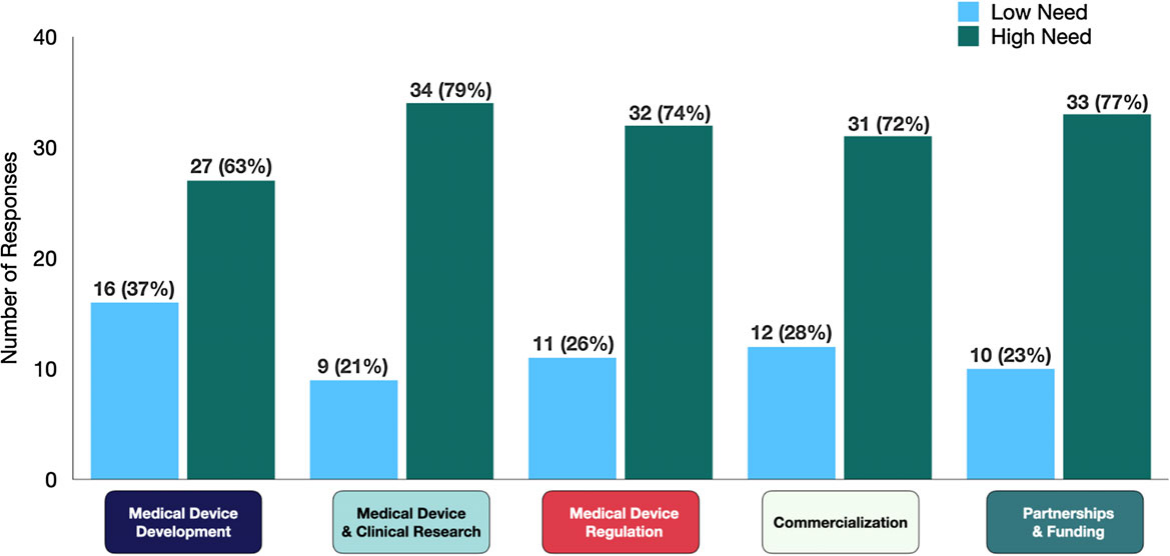
Educational barriers identified by startup innovators within five domains (n, %). The low need represents the combined responses under categories 1 and 2: “I do not need additional information,” or “I could use brief overview respectively,” and high need represents the combined responses under category 3 and 4: “I have background of topic but have specific questions” or “I need to develop an in-depth understanding, respectively.”

A majority of companies (53%) reported that they search for MDD-related information on a weekly basis, but they were somewhat satisfied with the information they found (58%). Respondents rated their interest on various topics on scale of 1–4 with 1 being no need of additional information and 4 being I need to develop an in-depth understanding. Medical device and clinical research topics were identified as the area of greatest need (categories 3 and 4) with 79% of company participants needing additional resources (Fig. 1).

### Investigator survey

**Figure 2.**
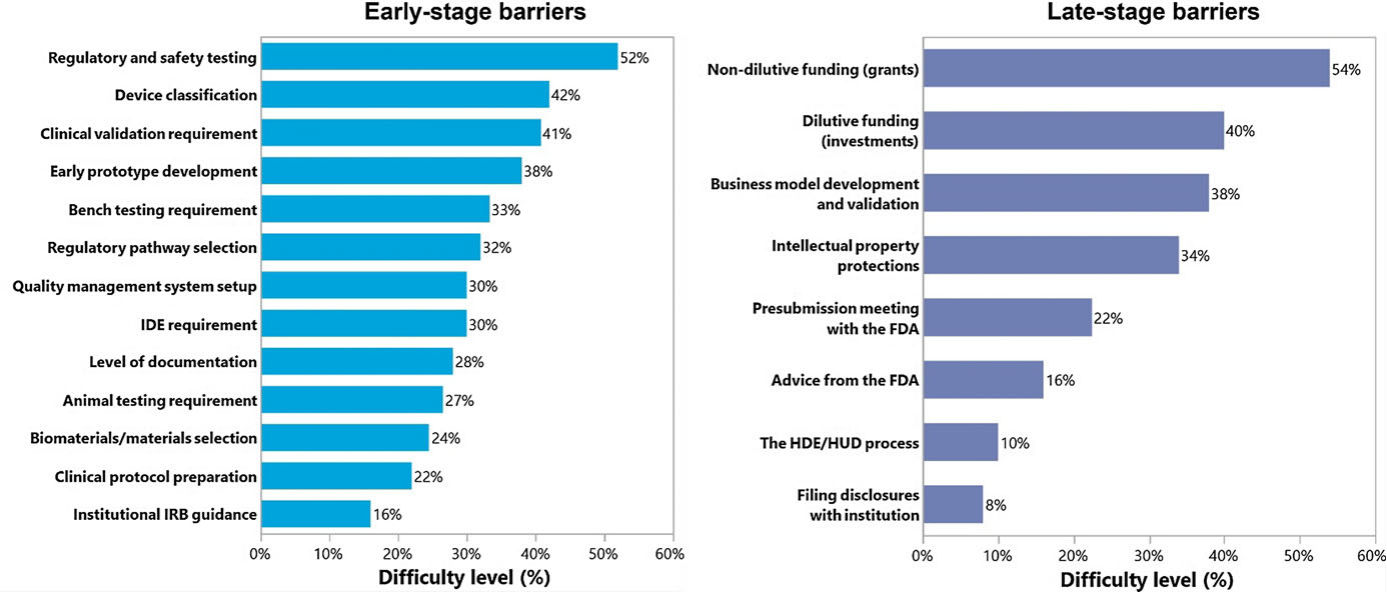
Pediatric medical device development barriers identified by academic investigators. The percentage represents the high need of resources under difficult and very difficult categories. FDA = United States Food and Drug Administration; HDE = Humanitarian Device Exemption; HUD = Humanitarian Use Device; IDA = Investigational Device Exemption; IRB = Institutional Review Board.

Fifty-two individuals completed the investigator survey, of which 79% had a faculty position, and 45% had a clinical background (Table 2). 92% of investigators were currently or previously involved with PMDD and 80% reported that their research led to an invention disclosure, patent, or other intellectual property for a medical device. The biggest barrier for investigators in the early stages was understanding regulatory and safety requirements testing with 52% of participants reporting it to be very difficult or somewhat difficult, while in the later stages, finding and obtaining non-dilutive funding was the most difficult for 54% of participants (Fig. 2). The largest experience gap for investigators was in setting up quality management systems and determining animal testing requirements (56 and 55% reporting no experience in this area, respectively).

**Table 2.** Demographics of investigator survey respondents

Category		*n*	%
*Academic position*			
	Assistant professor	13	25%
	Associate professor	19	37%
	Professor	9	17%
	Staff	3	6%
	Trainee	5	10%
	Other	3	6%
*Professional background*			
	Clinical	38	45%
	Engineering	23	27%
	Basic science	10	12%
	Computer science	4	5%
	Regulatory science	4	5%
	Legal	1	1%
	Other	5	6%

### Comparison of startups and investigator perspectives

**Figure 3.**
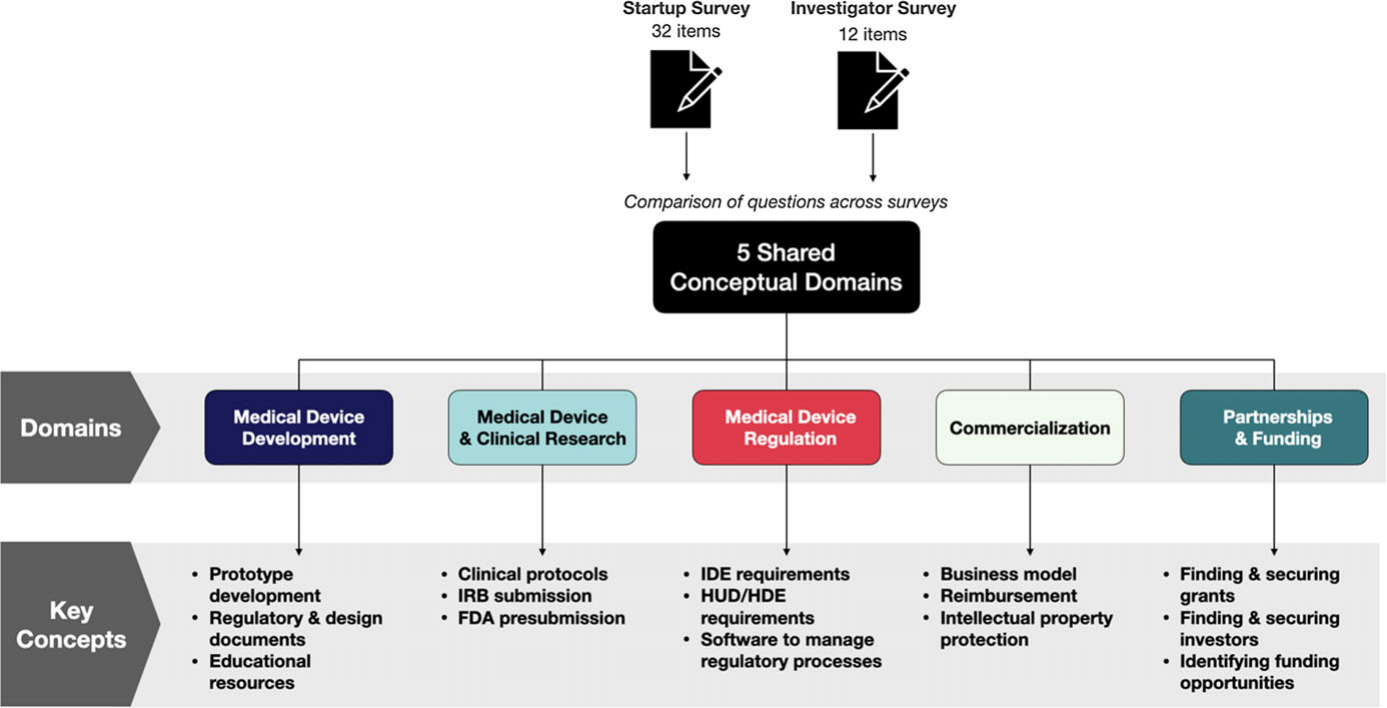
Concept mapping describing the 5 key domains in pediatric medical device development. FDA = United States Food and Drug Administration; HDE = Humanitarian Device Exemption; HUD = Humanitarian Use Device; IRB = Institutional Review Board.

The startup and Investigators surveys were administered at different times and featured different questions tailored to their respective audience, so to aggregate and compare responses, we developed a concept mapping between the two. Each question in the startup survey was matched to one to four questions in the investigator survey based on the overall concept that was being ascertained. We identified five key domains: medical device development, clinical and device research, medical device regulation, commercialization, and partnership and funding opportunities (Fig. 3). For example, in domain 1 Medical Device Development, we asked companies “Which topics would you like covered: Medical device development including concept and validation, design, prototyping, manufacturing, preclinical testing?;” whereas we asked investigators “How difficult has it been developing an early prototype of the device?,” “How difficult has it been determining the business model development and validation?” etc. The detailed mapping is described in Supplemental Table 2. Supplemental Tables 3, 4, and 5 show the barriers faced by startups and academic investigators individually.

### Domain 1: Medical device development

This domain includes the questions related to concept, early prototyping, design, validation, business model, and need for regulatory documents. 10% of startups reported that there are not enough resources for concept and prototype stages of TLPC. 63% of startups reported that they need either in-depth understanding of this topic or they had specific questions. Similarly, 38% of investigators reported that developing early prototyping and business model and validation was very difficult. 10% of investigators didn't have any experience of developing early prototyping. 79% of investigators reported that a library of educational resources specific to medical development would be moderately to extremely useful. 92% of investigators also agreed with the companies that templates for design documents would be useful.

### Domain 2: Medical device and clinical research

This domain includes the questions related to preclinical and clinical testing, Institutional Review Board (IRB) submission, writing clinical protocol, budgeting, and data qualities. 79% of company participants reported a high need for in-depth understanding of the topic and 33% reported not having enough educational resources for preclinical and clinical stages of TLPC. However, on the investigator side, investigators reported that writing clinical protocol (50%) and submitting an IRB application (62%) was easy. However, they reported that judgment for the bench testing was difficult (55%) and 28 participants didn't have experience with animal testing of their device.

### Domain 3: Medical device regulation

This domain includes the questions related to device classification and pathways-PMA, HDE/HUD submissions, software need for regulatory purposes, and presubmissions with the FDA. The majority (92%) of startups were familiar with their device classification but only 33% received the regulatory decision from FDA at the time of survey. 74% of company participants reported a high level of need for additional educational resources on this topic. Approx. half of investigators (42%) reported that identifying device classification and selection of appropriate regulatory pathway (32%) was difficult. The majority of investigators didn't have experience with the HDE/HUD process (82%), whereas 34% didn't have experience with determining IDE requirements. Investigators agreed with the companies on clinical research subtopics such as determining a presubmission with the FDA with 47% of investigators not having experience with that topic and 22% finding it difficult. 86% of investigators also reported that software to help guide and manage regulatory processes would be useful.

### Domain 4: Commercialization

This domain includes topics on entrepreneurship, marketing, business model, and customer discovery. 30% of startups felt that there were not enough educational resources on marketing and commercial use of pediatric medical devices, and 72% of them demanded more in-depth information on the topic. Investigators reported that obtaining intellectual property protections (36%) and filing disclosures with institutions (50%) seems easy. Half of investigators had difficulty in developing business models and validation (48%), and 22% did not have experience with it.

### Domain 5: Partnerships and funding opportunities

This domain was focused on partnership and funding opportunities with academia, industry, advocacy groups, investors, SBIR. Only 30% of startups were members of medical device advocacy or industry groups, and 77% of company participants reported a high level of need for additional educational resources. On the other hand, 98% of investigators reported that a list of funding opportunities for medical device development would be useful. Investigators found that obtaining non-dilutive funding (54%) and dilutive funding (40%) was difficult.

### Information-seeking behavior

Startup companies reported that the most common resources for advice were consultants, advisory board, and mentors followed by accelerators for domain 1(78%),2 (69%),4 (69%), and 5 (55%). While the FDA was reported as one of the most popular sources for medical device regulation domain (67%), it was among the least popular sources for the other domains. Non-profit organizations and the NIH were reported to be the least popular sources across all domains. On the investigators side, 17% investigators reported that they review FDA-guidance documents followed by their institution’s technology transfer office (TTO) (15%) and regulatory consultants (13%).

## Discussion

Despite the increase in the medical device market size, the sector is still dominated by adult medical devices [5]. The limited devices for pediatric health care delivery are attributed to fewer pediatric disease population, difficulty in clinical trial enrollment, parental consenting, and liability concerns [15]. Academic investigators and startups are two key sources of pediatric medical devices, but their knowledge gaps about the PMDD process have not been well characterized in the past. We conducted an educational needs assessment related to pediatric medical device development among startups and academic investigators. To compare both surveys, we created a concept map in five different domains: medical device development, medical device and clinical research, medical device regulation, commercialization, and partnership and funding opportunities [16]. This concept mapping helped to explain what areas need more attention in these two communities and how we can provide resources.

Investigators in academia and startups identified similar education needs in domain 1 medical device development, domain 3 medical device regulation, and domain 4 need for commercialization. The concept and prototyping stages are essential for bringing the product into commercialization in a timely fashion [16]. The FDA TPLC provides quality system guidance to a wide variety of companies and identification of regulatory compliance strategies. Innovators should review their innovative product design fits into the accepted definition of a medical device and fulfills all design specifications. Prototypes also play a critical role in obtaining quality feedback from end users and securing patents. Medical device regulation is another major area requiring educational resources. The innovators need to familiarize themselves with the three-tier device classification system and regulatory approvals [7]. Each device follows a specific pathway from manufacture to physician use and patient care depending on the assessment of risk associated with the device or classes of devices. There are no pediatric device-specific FDA review pathways adding additional complexity, but knowledge should be provided regarding Premarket Notification 510(k), De Novo Classification Request, Premarket Approval (PMA), and Humanitarian Device Exemption (HDE) pathways [7]. The FDA also has designation programs that can provide certain regulatory benefits to the device sponsor during the review phase, including Humanitarian Use Device (HUD) [17], Breakthrough Device Designation [18], and the Safer Technologies Program (STeP) [19]. None of these programs are pediatric-specific but have been used to advance pediatric devices. The FDA Presubmission is a way for companies to request feedback from the agency on potential and planned medical devices, but it tends to get underutilized. Under domain 4 commercialization, the majority of startups reported the need for educational resources. Although the success of commercialization is highly dependent on its precursor stages, innovators often fail at this stage. They should be educated to de-risk commercialization strategies by evaluating market size, how to protect IP, strategic partnerships, and leveraging the product to global market [4]. Investigators were able to find the resources easily for IP submissions and filing disclosures but identified the educational needs for business models and validation. Investigators stated the issue was *“Figuring out how to get started in translating the inventions/lab results into a business model for actual commercial development”* and reported *“a need for a robust educational system centered on customer development and business model canvas.”*

Companies need greater support in domain 2 medical device-related research such as preclinical and clinical testing, whereas for investigators there are several academic resources available such as the Clinical and Translational Science Awards Program [19] and TTO [21]. The program supports the investigators to test and develop innovative approaches to barriers in clinical research. For example, the efficient recruitment of research participants and IRB approvals for multisite clinical trials. Whereas the startups often struggle to identify the requirements for animal vs human testing, finding the institution for clinical trial set up, introduction with interested investigators, IRB submissions, and lack of knowledge understanding sponsor and investigator responsibilities for an efficient clinical trial. The other major issue is associated with the cost of each clinical study for data collection, patient enrollment, monitor adherence, providing interventional devices, and performing data analysis. Such traditional clinical trials can have strict inclusion and exclusion criteria that makes it challenging for researchers to accurately extrapolate the results to a broader population. To address this issue of generalizability, Real-World Evidence studies [22] can help researchers to understand how their products work. Patient cohorts can be identified using procedure or CPT/ICD codes and their healthcare utilization can be tracked longitudinally for better prognosis. Innovators can leverage this data for product innovation, to inform evidence-based pricing strategies, for business models, and to support regulatory requirements [23].

Investigators need more support in domain 5 partnership and funding opportunities to find non-dilutive (grants) and dilutive funding sources (investment). One of the investigators reported *“The small pilot grants/University innovation funds aren't enough to cover time + needs of developing the new device. Thus, at best, development moves very slowly.”* Another reported that *“The barrier is that it just takes much more time and work and therefore funding than anyone expects. There are many pilot funding opportunities but few keep-a-good-thing-going funding opportunities.”* One of the investigators reported finding partnership is a barrier *“Finding business partners is extremely difficult. Once you have a prototype, evaluating the market and finding adequate funding is almost too much for a clinical provider.”* Securing funding is an integral part of project execution but it is not well discussed in medical science, resulting in project failure [24]. To address this issue primarily, investigators can be assisted to choose the right study design with a small sample size that can be completed without external funding. If this is not the case the investigators should be educated in how to find the funding agencies and right funding mechanism. There are multiple funding sources such as local, national, and international funding bodies that can provide grants necessary for research and they all have different timelines. The FDA also addressed this gap by providing funding to the PDC program to provide pediatric device innovators with seed funding and expertise [9]. As stated by one of the investigators serving as PI of the study, “they need to fulfill many duties like teaching, publishing, and monitoring compliance; the institutions should provide additional education to prepare appealing research grants, appropriate budgets, and additional support to complete the submission in time” [24].

It is worth noting that the two groups of respondents had different backgrounds which likely influence their educational needs. Academic investigators were primarily clinicians (45%), engineers (27%), while the most common backgrounds for startup respondents were research (41%), entrepreneur (35%), and engineering (33%). A larger proportion of academic investigator respondents had a clinical background compared to the startup respondents (45% vs. 22%), which may explain why startups identified clinical research as a bigger educational need than academic investigators. Our findings highlight an opportunity to increase formal and informal early-stage collaboration between investigators. Organizations working to advance pediatric device development should understand who their primary learners are and plan their educational offerings accordingly. There are opportunities for different organizations to collaborate and share educational materials to improve efficiency and increase the efficiency of limited resources. The UCSF Stanford PDC assists projects through weekly innovators forums by providing expert feedback, personalized biodesign coaching, and advising [26]. CTIP provides learning opportunities through our online portal, monthly educational webinars, and forums to connect with other innovators to share experiences [27]. This needs assessment helps inform the topics and content covered through our various channels. The Regulatory Guidance for Academic Research of Drugs and Devices (ReGARDD) [28] is another resource developed for academia to cover some of the educational gaps. ReGARDD comprises a team of multi-institutional regulatory affairs specialists and experts to assist academic researchers in navigating an increasingly complex regulatory environment. CTIP also shares ReGARDD resources with startup for the development of successful strategies for medical device development. Academic institution and industry collaborations similar to The Pediatric Device Innovation Consortium model can also help address these educational gaps, knowledge transfer, and technological innovation [5,27,28]. Finally, public forums like the Pediatric Device Innovators Forum, a partnership between the FDA and the Pediatric Device Consortia, create an important platform to highlight critical issues in PMDD and discuss potential solutions [29].

### Limitations

The instruments from this paper are focused on academia and industry innovators. One of the limitations is it may not be generalizable to other populations but there are no validated instruments available to assess the barriers in PMDD. Future research is needed to validate these instruments in the general population and to identify the most effective way to meet the educational needs of these two communities. In this study, we reached out to the stakeholders of PDC program of FDA to identify the startups, and the investigator survey was limited to 4 academic institutions in the US West Coast that are part of active local innovation ecosystems. These responses may not be representative of other institutions or other geographic areas. Third-party interview services and anonymous data collection for a broader audience may reduce selection bias and generalizability. It is also possible that an individual may have completed both surveys, but highly unlikely given the different administration times, recruitment strategies, targeted participants, and survey instructions. Another limitation is we did not differentiate between pediatric vs adult devices, but it is rare for a company or investigator to have experience in both adult and pediatric device development. While none of the questions in the startup survey were specific to pediatrics, the survey itself came from CTIP, one of the FDA’s pediatric device consortia, and was targeted at only pediatric medical device companies, so the responses themselves reflect the needs of pediatric device development. Two surveys were designed and administered at different time points and for different audiences making it difficult to compare their results. Our concept mapping helped address this issue, but the associations were not always one-to-one, and so conclusions can only be drawn at a more generic level, such as the domain. Moreover, our survey included the option to provide additional input in the form of a free text field. We have presented in the discussion section several representative quotes, but a complete qualitative analysis was not performed.

## Conclusion

Between startup companies and academic investigators, there is a pool of similar, but not identical, needs to better understand the process of pediatric medical device development. Investigators need more information to identify the funding resources, whereas startup companies had an increased need for education on regulatory processes. These findings provide guidance on areas where CTIP and other support organizations can focus their education effort to advance pediatric medical device development by bridging key knowledge gaps.

## Supporting information

Shah et al. supplementary material Shah et al. supplementary material

## Supplementary material

The supplementary material for this article can be found at https://doi.org/10.1017/cts.2023.633.
