# Supplementary material for: PMDedu: Assessing the educational needs of startups and academic investigators focused on pediatric medical device development
Source: J Clin Transl Sci. 2023 Sep 25;7(1):e235. doi: 10.1017/cts.2023.633 (PMC10663766; doi:10.1017/cts.2023.633)
Supplement: Shah et al. supplementary material [file S2059866123006337sup001.docx]

- **Supplemental Table 1: Description of Device classification and Total Product Life Cycle**

| **Device classification** | | |
| --- | --- | --- |
| Device class | Examples | Regulatory process |
| Class-I | Mild to Moderate risk   - Oxygen mask - Electric toothbrush | Exempt from Premarket Notification (PMN/(510k)) and Premarket Approval (PMA) |
| Class-II | Moderate to high risk   - Surgical gloves - Catheters | PMN required |
| Class-III | High risk   - Pacemakers - Cochlear implants | PMA required |
| **Total Product Life Cycle** | | |
| Concept | The innovator makes certain that the innovative product design fits into the accepted definition of a medical device and the marketable use is clearly identified based on the available research and resources. | |
| Prototype | In this stage, the team tests the design specifications, validation, assesses the compliance with rules and regulations and product’s ability for reimbursement. Innovators also try to secure patents and intellectual property. | |
| Preclinical testing | During testing, the product goes through verification and validation processes, biocompatibility, and toxicology testing. This stage requires clinicians’ and engineers’ active involvement for design optimization to resolve conflict between user requirements and manufacturing capabilities. | |
| Clinical testing | This stage involves obtaining evidence for the evaluation of data about the safety and performance of medical products in their intended use. The clinical studies require Institutional Review Board (IRB) approval. | |
| Manufacturing | This stage is critical to assure that when design makes it to market, it fulfills promises of the functionalities. It involves clear, objective and written design inputs and design outputs such as drawings, specifications or other marketing considerations. | |
| Marketing | Once the medical product comes in the market, the innovators continue to collect user reported outcomes and any adverse events for post-marketing surveillance. | |
| Commercial Use | Innovators is working on strong business plans, marketing team possession of IP and regulatory approval. | |
| Obsolescence | In this stage, innovators should follow the guidelines to incorporate new clinical evidence if more effective devices enter the market and should inform the end-users about stopping the use of old devices. | |

- **Supplemental Table 2: Comparison of startups and investigator perspectives**

| **Domain** | **Company Question** | **Investigator question** |
| --- | --- | --- |
| Medical Device Development | CTIP is developing educational resources for pediatric medical device startups. Which topics would you like covered: Medical device development including concept and validation, design, prototyping, manufacturing, pre-clinical testing | How difficult has it been developing an early prototype of the device? |
|  |  | How difficult has it been determining the business model development and validation? |
|  |  | How useful would a library of educational resources specific to medical device development be? |
|  |  | How useful would templates of regulatory and design documents be? |
| Medical Device and Clinical Research | Which topics would you like to be covered through education activities?  Medical device and clinical research including: early feasibility trials, feasibility trials, pivotal trials, non-clinical studies, international trials, IRB, clinical study  design, research budget, implementation, recruitment, data quality, FDA audit | How difficult has it been to determine where to get help writing clinical protocol? |
|  |  | How difficult has it been to determine how to submit to their IRB? |
|  |  | How difficult has it been to determine scheduling, preparing for, and participating in a presubmission with the FDA? |
| Medical Device Regulation | Which topics would you like to be covered through educational activites? Medical device regulation including: FDA classification, Class I, Class II,  Class III, significant risk device, non-significant risk device, emergency use,  compassionate use, HDE, IDE, HUD, PMA, 510k, de novo, digital health  pathway, software as a medical device | How useful would software to help guide and manage regulatory process be? |
|  |  | How difficult has the HDE/HUD process been? |
|  |  | How difficult has it been to determine requirements for an IDE? |
| Commercialization | What topics would you like to be covered through educational activities:  Commercialization including: entrepreneurship, business model, intellectual property, market research, customer discovery, marketing, reimbursement | How difficult has it been obtaining appropriate intellectual property protections? |
| Partnerships and funding | What topics would you like to be covered through educational activities: Partnerships and funding opportunities: Academia, industry, patient advocate  groups, government agencies, investors, non-profits, SBIR/STTR | How difficult has it been finding and obtaining non-dilutive funding (grants)? |
|  |  | How difficult has it been finding and obtaining dilutive funding (investments)? |
|  |  | How useful would a list of funding opportunities specifically for medical device development be? |

- **Supplemental Table 3: Educational barriers identified by startup innovators (n=49)**

| **Domains** | **N (%)** |
| --- | --- |
| **Medical Device Development topic:**  **Concept, design, manufacturing, pre-clinical testing** | |
| I don’t need additional information | 6 (12) |
| I could use brief overview | 10 (20) |
| I have background of topic but have specific questions | 20 (41) |
| I need to develop an in-depth understanding | 7 (14) |
| **Medical device and clinical research topic:**  **Clinical studies, IRB, budget, recruitment, audit, data quality** | |
| I don’t need additional information | 5 (10) |
| I could use brief overview | 4 (8) |
| I have background of topic but have specific questions | 16 (33) |
| I need to develop an in-depth understanding | 18 (37) |
| **Medical device regulation topic:**  **Class I, II, III, and other pathways** | |
| I don’t need additional information | 4 (8) |
| I could use brief overview | 7 (14) |
| I have background of topic but have specific questions | 18 (37) |
| I need to develop an in-depth understanding | 14 (29) |
| **Commercialization topic:**  **Entrepreneurship, marketing, business model, customer discovery** | |
| I don’t need additional information | 4 (8) |
| I could use brief overview | 8 (16) |
| I have background of topic but have specific questions | 13 (27) |
| I need to develop an in-depth understanding | 18 (37) |
| **Partnership and Funding opportunities topic:**  **Academia, Industry, advocacy group, investors, SBIR** | |
| I don’t need additional information | 3 (6) |
| I could use brief overview | 7 (14) |
| I have background of topic but have specific questions | 20 (41) |
| I need to develop an in-depth understanding | 13 (27) |

- **Supplemental Table 4: Early-stage medical device development barriers identified by academic investigators (n=50).**

| **#** | **Question** | **No Experience**  **N (%)** | **Easy**  **N (%)** | **Difficult N (%)** |
| --- | --- | --- | --- | --- |
| 1 | Understanding the regulatory and safety testing requirements for my type of device | 6 (12) | 7 (14) | 26 (52) |
| 2 | Identifying the proper device classification | 5 (10) | 17 (34) | 21 (42) |
| 3 | Selecting an appropriate regulatory pathway (e.g., 510(k), De Novo, PMA) | 8 (16) | 17 (34) | 16 (32) |
| 4 | Biomaterials/materials selection | 16 (33) | 9 (18) | 12 (24) |
| 5 | Developing an early prototype of my device | 5 (10) | 17 (34) | 19 (38) |
| 6 | Appropriate level of bench testing required at each stage of development | 10 (21) | 8 (16) | 16 (32) |
| 7 | Requirements for animal testing of my device(s) | 27 (55) | 4 (8) | 13 (27) |
| 8 | Setting up a quality management system | 28 (56) | 1 (2) | 15 (30) |
| 9 | The appropriate level of documentation for each phase of development | 20 (40) | 3 (6) | 14 (28) |
| 10 | Requirements for clinical validation of my device | 13 (27) | 11 (22) | 20 (41) |
| 11 | Requirement for an IDE (i.e., is it a significant risk device?) | 17 (34) | 10 (20) | 15 (30) |
| 12 | Where to get help writing a clinical protocol | 8 (16) | 25 (50) | 11 (22) |
| 13 | How to submit to my institutional IRB | 6 (12) | 33 (62) | 8 (16) |

- **Supplemental Table 5: Late-stage medical device development barriers identified by academic investigators (n=50).**

| **#** | **Question** | **No Experience**  **N (%)** | **Easy**  **N (%)** | **Difficult N (%)** |
| --- | --- | --- | --- | --- |
| 1 | Scheduling, preparing for, and participating in a presubmission meeting with the FDA | 23 (47) | 9 (18) | 11 (22) |
| 2 | Seeking advice or clarification from the FDA | 25 (50) | 12 (24) | 8 (16) |
| 3 | The HDE/HUD process | 41 (82) | 1 (2) | 5 (10) |
| 4 | Filing disclosures with my institution | 12 (24) | 25 (50) | 4 (8) |
| 5 | Obtaining appropriate intellectual property protections | 7 (14) | 18 (36) | 17 (34) |
| 6 | Business model development and validation | 11 (22) | 7 (14) | 19 (38) |
| 7 | Finding and obtaining non-dilutive funding (grants) | 8 (16) | 10 (20) | 27 (54) |
| 8 | Finding and obtaining dilutive funding (investments) | 24 (48) | 4 (8) | 20 (40) |
